# Health technology assessment of drugs for rare diseases: insights, trends, and reasons for negative recommendations from the CADTH common drug review

**DOI:** 10.1186/s13023-016-0539-3

**Published:** 2016-12-01

**Authors:** Ghayath Janoudi, William Amegatse, Brendan McIntosh, Chander Sehgal, Trevor Richter

**Affiliations:** CADTH, 865 Carling Ave., Suite 600, Ottawa, ON K1S 5S8 Canada

**Keywords:** Rare diseases, Orphan drugs, Technology assessment, health, Canada

## Abstract

**Background:**

A shift in biochemical research towards drugs for rare diseases has created new challenges for the pharmaceutical industry, government regulators, health technology assessment agencies, and public and private payers. In this article, we aim to comprehensively review, characterize, identify possible trends, and explore reasons for negative reimbursement recommendations in submissions made to the Common Drug Review (CDR) for drugs for rare diseases (DRD) at the Canadian Agency for Drugs and Technologies in Health (CADTH), a publicly funded pan-Canadian health technology assessment agency. A public database (cadth.ca) was screened to identify DRD submissions to CDR. A diseases prevalence of ≤50 per 100,000 people was considered a rare disease. We calculated descriptive statistics for prevalence, study design, study size, treatment cost, reimbursement recommendation types, and reasons for negative reimbursement recommendations.

**Results:**

From 2004 to 2015, 63 of 434 submissions to the CDR were for DRD (range: 1 submission in 2005 to 10 submissions in 2013). Most (74.6%) submissions included at least one double-blind randomized controlled trial (RCT). The average study size was 190 patients (range: 20 to 742). The average annual treatment cost was C$215,631 (range: $9,706 to $940,084). Reimbursement recommendations were positive for 54% of the submissions. Negative reimbursement recommendations were made due to a lack of clinical effectiveness (38.5%), insufficient evidence (30.8%), multiple reasons (23.1%), or lack of cost effectiveness/high cost (7.7%).

**Conclusion:**

The number of DRD submissions to CDR increased since 2013; from 4 to 5 per year between 2004 and 2012, to 10, 9, and 8 in 2013, 2014, and 2015 respectively. More than half of DRD submissions received positive reimbursement recommendation. Poor quality evidence and/or lack of supportive clinical evidence was at least partly responsible for a negative reimbursement recommendation in all cases. Although the average cost of DRD treatments was high, high cost was a reason for a negative reimbursement recommendation in only two (7.7%) of negative reimbursement recommendations.

## Background

The pipeline of drugs for rare diseases (DRD) has been increasing as biochemical research has shifted focus from blockbusters to ‘niche’ drugs [1]. While all rare diseases, by definition, affect relatively small populations, the exact number of patients affected and the extent of the severity of the condition used to define a rare disease is a matter of continuous debate [2]. The regulator in Canada, Health Canada, is yet to officially adopt a definition of a rare disease, although in a 2014 press release, Health Canada defined a rare disease as a “life-threatening, seriously debilitating, or serious chronic condition that only affects a very small number of patients (typically less than 5 in 10,000 persons)” [3].

The Canadian Agency for Drugs and Technologies in Health (CADTH) is a publicly funded Canadian health technology assessment (HTA) organization that evaluates drugs for reimbursement purposes through its Common Drug Review (CDR) and pan-Canadian Oncology Drug Review [pCODR] processes [4]. Through its advisory body, the Canadian Drug Expert Committee (CDEC), CADTH offers non-binding reimbursement recommendations to all public drug plans in Canada except for those in Quebec, which has a separate review process. Overall, CDEC provides reimbursement recommendations under three themes: List, List with clinical criteria or conditions, and do not list. It has been reported that participating drug plans adhere to these recommendations in more than 90% of formulary listings [4]. In 2012, CADTH received a formal mandate from its federal, provincial, and territorial (F/P/T) funders to review DRDs [5]. Prior to 2012, there was no formal distinction given for DRDs and CADTH evaluated such drugs under the same process as non-DRDs. After a formal stakeholder consultation, CADTH decided to keep the evaluation of DRDs under an enhanced CDR process, with more emphasis on engagement of specialists, patient input, and offering opportunities for manufacturers to engage in dialogue with CADTH earlier in the pre-submission phase [6]. As such, CADTH has no formal definition that would make a distinction between DRDs and non-DRDs.

This report provides a summary of key characteristics for all DRD submissions that have been filed for review through the CDR process, an analysis of the volume and frequency of DRD submissions, and a detailed examination of the reimbursement recommendations and reasons for reimbursement recommendation that have been issued for DRDs. This will allow us to draw a bigger picture of the fast evolving landscape of DRDs, explore the characters of DRDs CDR submissions, and to gain insight into the evidence base that supports HTA process for DRDs.

## Methods

On February 3, 2016, CDR reimbursement recommendation reports published on the CADTH website (www.cadth.ca) were screened for inclusion in this review. A drug was included if it was reviewed for use in the management of a disease with a prevalence or birth prevalence of less than 50 in 100,000 people. This definition of rare diseases is in line with most international jurisdictions [2]. Two independent reviewers (G.J. and W.A.) screened the retrieved reports. First-level screening excluded CDR reports with indications for diseases that are not rare. Second-level screening required the reviewer to provide the prevalence of the disease before a decision to exclude or include was made. Discrepancy between the two reviewers was resolved through arbitration of a third independent reviewer (T.R.). One reviewer (G.J.) extracted the following data from the published CDR reports: CADTH project ID, project status, date of submission, type of submission, disease under review, prevalence of disease under review, Health Canada indication, manufacturer name, drug brand name, drug non-proprietary name, number of studies considered within the CDR review, type of studies considered in each submission, comparisons used in the studies, size of the largest study, highest evidence level (with no double counting allowed), per patient annual treatment cost, cost per quality-adjusted life year (QALY), reimbursement recommendation, and reason(s) for a negative reimbursement recommendation. If prevalence data were not reported in the CDR report, we sought a Canadian published estimate in the literature, if such estimate could not be found, then the prevalence point estimates was informed from the Orphanet report series [7]. Full data-set is available from the corresponding author upon reasonable request.

We categorized reimbursement recommendations as either “List”, “Do Not List”, or “List with Conditions/Criteria”. A reimbursement recommendation of “do not list at the submitted price” was classified as “Do Not List”. We thematically explored reasons for a negative reimbursement recommendation (i.e., a “Do Not List”) based on the most prominent reason stated within the recommendation report, and each reason was categorized as one of the following:

- Insufficient evidence: in cases where the committee found the evidence to be of poor quality with a high degree of statistical uncertainty and methodological limitations
- Lack of clinical effectiveness: in cases where the committee could not determine the actual clinical value to be observed in practice due to a lack of (a) relevant clinical outcomes, (b) knowledge of what constituted a minimal clinically important difference (MCID), or (c) the use of outcomes tha﻿t are not validated. Usually, in this category, high-quality studies would have been considered, and the drug would have potentially shown statistical significance in possible surrogate outcomes
- Lack of cost-effectiveness/high cost: in cases where the committee determined the treatment price to be contextually unacceptable
- Multiple reasons: in cases where more than one of the aforementioned reasons was applicable

Due to the implementation of a revised reimbursement recommendation framework at the CADTH CDR in November 2012 [8], a subgroup analysis of CDEC reimbursement recommendations that were issued after October 2012 were further analyzed separately. The accuracy of the extracted data was verified by a second reviewer (T.R.).

We calculated descriptive statistics of various parameters, including the average, standard deviation (SD), 95% confidence interval (95%CI), median, and range of continuous variables. Categorical variables are presented as percentages.

## Results

**Fig. 1:**
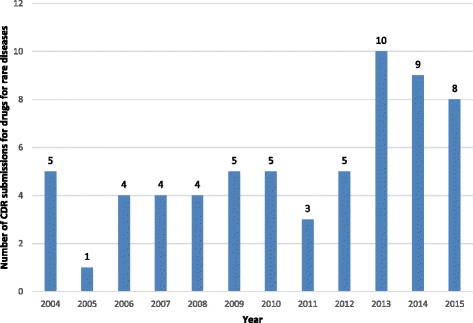
Number of CDR submissions for drugs for rare diseases per year for the period 2004 to 2015. Numbers above columns indicate the number of submissions

Of 434 records retrieved on February 3, 2016, we identified 63 submissions for DRDs. Of these 63 submissions, four were ongoing at the time of the search, and the reimbursement recommendations and clinical report were not available. Therefore, there was no information available for these DRDs regarding the studies included in the submission, the price, and reimbursement recommendations. Assessment of the number of DRD submissions made to the CADTH CDR revealed two distinct, contrasting periods: the first period was between 2004 and 2012, within which the number of submissions was generally between 4 and 5 per year, and ranged from a minimum of 1 submission in 2005 to a maximum of 5 submissions in each of the years 2004, 2009, 2010, and 2012; the second period was between 2013 and 2015, which was distinct from the preceding period in that the number of submissions increased, up to 10, 9, and 8 submissions for the years 2013, 2014, and 2015, respectively. Figure 1 illustrates the temporal trend in the volume of DRD submissions.

Table 1 lists the date of each submission, along with the drug name, manufacturer name, the rare disease for which the drug is indicated, the prevalence of the disease, and the CDEC reimbursement recommendation. Examination of these data revealed that Pfizer Canada made more DRD submissions than any other pharmaceutical manufacturer (a total of six submissions), followed by Genzyme Canada (five submissions), and Alexion, Hoffman-La Roche, and Novartis (four submissions each).

**Table 1:** CDR for rare diseases

Date of submission	Drug brand name	Drug non-proprietary name	Manufacturer	Pharmacological category	Disease	Prevelance of disease (/100,000)	CDEC reimbursement recommendation
19/02/2004	Replagal	agalsidase alfa	Transkaryotic Therapies Inc.	Biologic/Enzyme replacement therapy	Fabry Disease	0.22^a^	Do not list
24/02/2004	Fabrazyme	agalsidase beta	Genzyme Canada Inc.	Biologic/Enzyme replacement therapy	Fabry Disease	0.22^a^	Do not list
14/07/2004	Remodulin	Treprostinil sodium	Northern Theraputics Inc.	Chemical/Vasodilating Agents	Pulmonary arterial hypertension	3.3^a^	Do not list
24/11/2004	Zavesca	miglustat	Actelion Pharmaceuticals Canada Inc.	Chemical/Substrate reduction therapy, Enzyme Inhibitor; Glucosylceramide Synthase Inhibitor	Gaucher disease	1^a^	Do not list
10/12/2004	Fabrazyme	agalsidase beta	Genzyme Canada Inc.	Biologic/Enzyme replacement therapy	Fabry Disease	0.22^a^	Do not list
03/02/2005	Aldurazyme	laronidase	Genzyme Canada Inc.	Biologic/Enzyme replacement therapy	Mucopolysaccharidosis I	8^a^	Do not list
25/01/2006	Somavert	pegvisomant	Pfizer Canada Inc.	Biologic/Growth hormone receptor antagonist	Acromegaly	5.5^a^	Do not list
24/02/2006	Remodulin	Treprostinil sodium	Northern Theraputics Inc.	Chemical/Vasodilating Agents	Pulmonary arterial hypertension	3.3^a^	List with criteria/condition
20/07/2006	Sutent	sunitinib	Pfizer Canada Inc.	Chemical/Antineoplastic Agent, Tyrosine Kinase Inhibitor; Antineoplastic Agent, Vascular Endothelial Growth Factor (VEGF) Inhibitor	Gastrointestinal stromal tumour	13^a^	List with clinical criteria and/or conditions
10/10/2006	Myozyme	alglucosidase	Genzyme Canada Inc.	Biologic/Enzyme replacement therapy	Glycogen storage disease type II	0.8^a^	List with clinical criteria and/or conditions
20/02/2007	Somatuline	lanreotide acetate	Ipsen Limited	Chemical/Somatostatin Analog	Acromegaly	5.5^a^	List in a similar manner to other drugs in class
22/06/2007	Thelin	Sitaxsentan sodium	Encysive Canada Inc.	Chemical/Endothelin receptor antagonist	Pulmonary arterial hypertension	3.3^a^	Do not list
13/07/2007	Elaprase	idursulfase	Shire Human Genetic Therapies (Canada) Inc.	Biologic/Enzyme replacement therapy	Mucopolysarccharidosis II	6.7^a^	Do not list
29/08/2007	Xyrem	sodium oxybate	Valeant Canada Ltd.	Chemical/Central Nervous System Depressant	Narcolepsy	25^a^	Withdrawn
05/05/2008	Thelin	Sitaxsentan sodium	Encysive Canada Inc.	Chemical/Endothelin receptor antagonist	Pulmonary arterial hypertension	3.3^a^	Do not list
09/07/2008	Xyrem	sodium oxybate	Valeant Canada Ltd.	Chemical/Central Nervous System Depressant	Narcolepsy	25^a^	Do not list
09/07/2008	Volibris	Ambrisentan	GlaxoSmithKline	Chemical/Endothelin receptor antagonist	Pulmonary arterial hypertension	3.3^a^	List with clinical criteria and/or conditions
29/08/2008	Orencia	Abatacept	Bristol-Myers Squibb Canada	Biologic/Selective T Cell Costimulation Modulator	Juvenile idiopathic arthritis (JIA)	44.7^b^	List with criteria/condition
12/06/2009	Omnitrope	somatropin (rDNA origin)	Sandoz Canada Inc.	Biologic/Growth hormone	Growth hormone deficiency in children and adults	37^c^	List in a similar manner to other drugs in class
12/06/2009	Xeomin	clostridium botulinum neurotoxin type A, free of complexing proteins	Merz Pharmaceuticals GmbH	Biologic/Neuromuscular Blocker Agent, Toxin; Ophthalmic Agent, Toxin	Blepharospasm	16.43^d^	List in a similar manner to other drugs in class
12/06/2009	Xeomin	clostridium botulinum neurotoxin type A, free of complexing proteins	Merz Pharmaceuticals GmbH	Biologic/Neuromuscular Blocker Agent, Toxin; Ophthalmic Agent, Toxin	Cervical dystonia	16.43^d^	List in a similar manner to other drugs in class
18/09/2009	Soliris	eculizumab	Alexion Pharmaceuticals Inc.	Biologic/Monoclonal Antibody; Monoclonal Antibody, Complement Inhibitor	Paroxysmal nocturnal hemoglobinuria	0.2^a^	Do not list
29/09/2009	Nplate	Romiplostim	Amgen Canada Inc.	Biologic/Colony Stimulating Factor; Hematopoietic Agent; Thrombopoietic Agent	Immune thrombocytopenic purpura	25^a^	Do not list
05/02/2010	Adcirca	Tadalafil	Eli Lilly Canada Inc.	Chemical/Enzyme inhibitor	Pulmonary arterial hypertension	3.3^a^	List with criteria/condition
07/07/2010	Ilaris	canakinumab	Novartis Pharmaceuticals Canada Inc.	Biologic/Interleukin-1 Beta Inhibitor; Interleukin-1 Inhibitor; Monoclonal Antibody	Cryopyrin-Associated Periodic Syndrome (CAPS)	0.3^e^	Do not list
08/07/2010	Kuvan	sapropterin dihydrochloride	BioMarin Pharmaceutical (Canada) Inc.	Chemical/Enzyme Cofactor, activates residual PAH enzyme	Phenylketonuria	10^a^	Do not list
28/10/2010	Vpriv	velaglucerase alfa	Shire Human Genetic Therapies (Canada) Inc.	Biologic/Enzyme replacement therapy	Gaucher disease	1^a^	List with clinical criteria and/or conditions
02/12/2010	Cayston	aztreonam	Gilead Sciences Canada Inc.	Chemical/Antibiotic	Cystic fibrosis	7.4^a^	List with clinical criteria and/or conditions
26/04/2011	Revolade	Eltrombopag olamine	GlaxoSmithKline	Chemical/Colony Stimulating Factor; Hematopoietic Agent; Thrombopoietic Agent	Immune thrombocytopenic purpura	25^a^	Do not list
01/06/2011	Kuvan	sapropterin dihydrochloride	BioMarin Pharmaceutical (Canada) Inc.	Chemical/Enzyme Cofactor, activates residual PAH enzyme	Phenylketonuria	10^a^	NR
20/09/2011	Banzel	Rufinamide	Eisai Limited	Chemical/Anticonvulsant, Triazole Derivative	Lennox-Gastaut Syndrome	15^a^	List with clinical criteria and/or conditions
24/01/2012	Mozobil	plerixafor injection	Genzyme Canada Inc.	Chemical/Hematopoietic Agent; Hematopoietic Stem Cell Mobilizer	Non-Hodgkin’s lymphoma and multiple myeloma	11.9^a^	Do not list
30/01/2012	Actemra	tocilizumab	Hoffmann-La Roche Ltd.	Biologic/Antirheumatic, Disease Modifying; Interleukin-6 Receptor Antagonist	Juvenile idiopathic arthritis	44.7^b^	List
24/02/2012	Rituxan	Rituximab	Hoffmann-La Roche Ltd.	Biologic/Antineoplastic Agent, Anti-CD20; Antineoplastic Agent, Monoclonal Antibody; Antirheumatic, Miscellaneous; Immunosuppressant Agent; Monoclonal Antibody	Granulomatosis with Polyangiitis	9^a^	List with clinical criteria and/or conditions
27/09/2012	Kalydeco	ivacaftor tablets	Vertex Pharmaceuticals (Canada) Inc.	Chemical/Cystic Fibrosis Transmembrane Conductance Regulator Potentiator	Cystic fibrosis	7.4^a^	List with criteria/condition
05/10/2012	Esbriet	pirfenidone	InterMune Canada Inc.	Chemical/Anti-inflammatory Agent; Antifibrotic Agent	Idiopathic pulmonary fibrosis (IPF)	42.7^f^	Do not list
07/01/2013	Soliris	eculizumab	Alexion Pharmaceuticals Inc.	Biologic/Monoclonal Antibody; Monoclonal Antibody, Complement Inhibitor	Atypical Hemolytic Uremic Syndrome	0.85^a^	Do not list
04/02/2013	Humira	adalimumab	AbbVie Corporation	Biologic/Antirheumatic, Disease Modifying; Gastrointestinal Agent, Miscellaneous; Monoclonal Antibody; Tumor Necrosis Factor (TNF) Blocking Agent	Juvenile idiopathic arthritis (JIA)	44.7^b^	List with clinical criteria and/or conditions
08/03/2013	Afinitor	Everolimus	Novartis Pharmaceuticals Canada Inc.	Chemical/Antineoplastic Agent, mTOR Kinase Inhibitor; Immunosuppressant Agent; mTOR Kinase Inhibitor	Tuberous sclerosis	8.8^a^	Do not list
27/05/2013	Genotropin GHD-A	somatropin	Pfizer Canada Inc.	Biologic/Growth Hormone	Growth hormone deficiency	37^c^	List with clinical criteria and/or conditions
27/05/2013	Genotropin GHD-P	somatropin	Pfizer Canada Inc.	Biologic/Growth Hormone	Growth hormone deficiency	37^c^	List with clinical criteria and/or conditions
27/05/2013	Genotropin TS	somatropin	Pfizer Canada Inc.	Biologic/Growth Hormone	Turner syndrome	50^g^	List with criteria/condition
06/06/2013	Jetrea	ocriplasmin	Alcon Canada Inc.	Biologic/Ophthalmic Agent; Vitreolytic	Vitreomacular adhesion	40^h^	List with criteria/condition
29/07/2013	Actemra	tocilizumab	Hoffmann-La Roche Ltd.	Biologic/Antirheumatic, Disease Modifying; Interleukin-6 Receptor Antagonist	Juvenile idiopathic arthritis (JIA)	44.7^b^	List with criteria/condition
30/09/2013	Adempas	Ricoiguat	Bayer Inc.	Chemical/Soluble Guanylate Cyclase (sGC) Stimulator	Pulmonary arterial hypertension	3.3^a^	List with criteria/condition
16/12/2013	Opsumit	Macitentan	Actelion Pharmaceuticals Canada Inc.	Chemical/Endothelin Receptor Antagonist; Vasodilator	Pulmonary arterial hypertension	3.3^a^	List with criteria/condition
27/02/2014	Signifor	pasireotide	Novartis Pharmaceuticals Canada Inc.	Chemical/Somatostatin Analog	Cushing's Disease	4^a^	Do not list
04/03/2014	Firazyr	icatibant	Shire Human Genetic Therapies (Canada) Inc.	Chemical/Selective Bradykinin B2 Receptor Antagonist	Hereditary angioedema	1^a^	List with clinical criteria and/or conditions
19/03/2014	Afinitor	Everolimus	Novartis Pharmaceuticals Canada Inc.	Chemical./Antineoplastic Agent, mTOR Kinase Inhibitor; Immunosuppressant Agent; mTOR Kinase Inhibitor	Tuberous Sclerosis	8.8^a^	Do not list
01/05/2014	Kalydeco	ivacaftor	Vertex Pharmaceuticals (Canada) Inc,	Chemical/Cystic Fibrosis Transmembrane Conductance Regulator Potentiator	Cystic fibrosis	7.4^a^	List with criteria/condition
08/07/2014	Juxtapid	lomitapide	Aegerion Pharmaceuticals (Canada) Ltd.	Chemical/Antilipemic Agent, Microsomal Triglyceride Transfer Protein (MTP) Inhibitor	Homozygous Familial Hypercholesterolemia	0.6^i^	Do not list
05/08/2014	Vimizim	elosulfase alfa	BioMarin Pharmaceutical (Canada) Inc.	Biologic/Enzyme replacement therapy	Mucopolysaccharidosis IV	0.3^j^	Do not list
29/08/2014	Esbriet	pirfenidone	Hoffmann-La Roche Ltd.	Chemical/Anti-inflammatory Agent; Antifibrotic Agent	Idiopathic pulmonary fibrosis (IPF)	42.7^f^	List with criteria/condition
15/09/2014	Elelyso	Taliglucerase alfa	Pfizer Canada Inc.	Biologic/Enzyme replacement therapy	Gaucher disease	1^a^	Do not list
30/10/2014	Diacomit	Stiripentol	Biocodex	Chemical/Anticonvulsant	Dravet Syndrome	2.5^a^	List with criteria/condition
09/02/2015	Soliris	eculizumab	Alexion Pharmaceuticals Inc.	Biologic/Monoclonal Antibody; Monoclonal Antibody, Complement Inhibitor	Atypical Hemolytic Uremic Syndrome	0.85^a^	Do not list
23/04/2015	Ofev	Nintedanib	Boehringer Ingelheim (Canada) Ltd.	Chemical/Tyrosine Kinase Inhibitor	Idiopathic pulmonary fibrosis (IPF)	42.7^f^	List with criteria/condition
08/05/2015	Kalydeco	Ivacaftor	Vertex Pharmaceuticals (Canada) Inc.	Chemical/Cystic Fibrosis Transmembrane Conductance Regulator Potentiator	Cystic fibrosis	7.4^a^	List with criteria/condition
25/06/2015	Adempas	Riociguat	Bayer Inc.	Chemical/Soluble Guanylate Cyclase (sGC) Stimulator	Pulmonary arterial hypertension	3.3^a^	List with criteria/condition
20/07/2015	Strensiq	Asfotase alfa	Alexion Pharmaceuticals Inc.	Biologic/Enzyme replacement therapy	Hypophophatasia	1^k^	Ongoing
20/08/2015	Naglazyme	Galsulfase	BioMarin Pharmaceutical (Canada) Inc.	Biologic/Enzyme replacement therapy	Mucopolysaccharidosis VI	0.3^j^	Ongoing
24/11/2015	Ilaris	canakinumab	Novartis Pharmaceuticals Canada Inc.	Biologic/Interleukin-1 Beta Inhibitor; Interleukin-1 Inhibitor; Monoclonal Antibody	Juvenile idiopathic arthritis	44.7^b^	Ongoing
27/11/2015	Orkambi	lumacaftor/ivacaftor	Vertex Pharmaceuticals (Canada) Inc.	Chemical/CFTR combinations	Cystic fibrosis	7.4^a^	Ongoing

Sources of prevalence estimates

^a^Orphanet Report Series - Prevalence of rare diseases: Bibliographic data - July 2015 - Number 2. http://www.orpha.net/orphacom/cahiers/docs/GB/Prevalence_of_rare_diseases_by_decreasing_prevalence_or_cases.pdf. Last accessed January, 2016

^b^Thierry S, Fautrel B, Lemelle I, Guillemin F. Prevalence and incidence of juvenile idiopathic arthritis: a systematic review. Joint Bone Spine. 2014 Mar;81(2):112–7. doi: 10.1016/j.jbspin.2013.09.003. Epub 2013 Nov 6

^c^Stochholm K, Gravholt CH, Laursen T, Jorgensen JO, Laurberg P, Andersen M. Incidence of GH deficiency - a nationwide study. Eur J Endocrinol. 2006 Jul. 155(1):61-71

^d^Steeves TD, Day L, Dykeman J, Jette N, Pringsheim T. The prevalence of primary dystonia: a systematic review and meta-analysis. Mov Disord. 2012 Dec;27(14):1789–96. doi: 10.1002/mds.25244. Epub 2012 Oct 31

^e^Kümmerle-Deschner JB. [Cryopyrin-associated periodic syndrome] [Article in German]. Z Rheumatol. 2012 Apr;71(3):199–208. doi: 10.1007/s00393-011-0856-9

^f^Ganesh Raghu, Derek Weycker, John Edelsberg, Williamson Z. Bradford, Gerry Oster. Incidence and Prevalence of Idiopathic Pulmonary Fibrosis. Am J Respir Crit Care Med Vol 174. pp 810–816, 2006

^g^Claus Højbjerg Gravholt, Kirstine Stochholm. The epidemiology of Turner syndrome. International Congress Series. Volume 1298, October 2006, Pages 139–145

^h^Ocriplasmin (Jetrea) (125 mcg intravitreal injection) CADTH Common Drug Review Clinical Review Report. January 2014. Available https://www.cadth.ca/sites/default/files/cdr/clinical/SR0337_Jetrea_CL_Report_e.pdf

^i^lomitapide (Juxtapid) (oral capsules) CADTH Common Drug Review Clinical Review Report. July 2015. Available https://www.cadth.ca/sites/default/files/cdr/clinical/SR0386_Juxtapid_CL_Report_e.pdf

^j^Lowry, R.B., Applegarth, D.A., Toone, J.R. et al. An update on the frequency of mucopolysaccharide syndromes in British Columbia. Hum Genet (1990) 85: 389. doi:10.1007/BF00206770

^k^Fraser D. Hypophosphatasia. Am J Med, 1957; 730–746

**Fig. 2:**
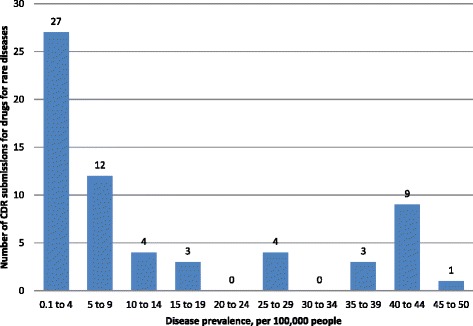
Number of CDR submissions for drugs for rare diseases by indicated disease prevalence. Numbers above columns indicate the number of submissions

Of the 63 DRD submissions, approximately half (30 or 47.6%) were for drugs that were classified by Health Canada as biologics, with the remainder being small molecules. The average prevalence of diseases for which DRD submissions were reviewed at CDR was 14.0 patients per 100,000 people (SD: 16.0; 95%CI: 9.9 to 18.0; range: 0.2 to 50.0 per 100,000 people; median: 7.4 per 100,000 people). Figure 2 presents the number of submissions by prevalence. Figure 2 illustrates that more than half of all DRD submissions (61.9%) were for treatments for rare diseases with a prevalence of less than 10 patients per 100,000 people, with the remainder of the DRD submissions (38.1%) being for treatments for diseases with a prevalence between 10 and 50 patients per 100,000 people. This suggests that there is a tendency for DRD submissions to target relatively less prevalent rare diseases.

**Fig. 3:**
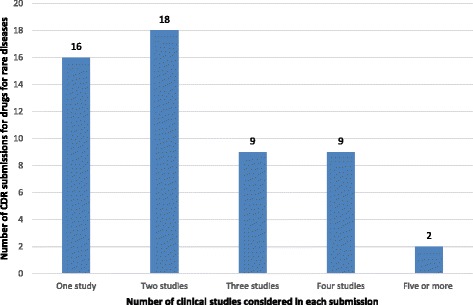
Number of CDR submissions for drugs for rare diseases by the number of clinical studies considered in each submission. Numbers above columns indicate the number of submissions

**Fig. 4:**
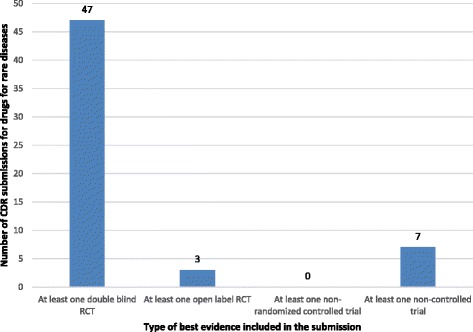
Type of best evidence included in the CDR submissions of drugs for rare diseases. Numbers above columns indicate the number of submissions

Of the 54 (85.7%) submissions in which the number of clinical studies considered by CDEC was reported, the average number of clinical studies per submission was 2.5 (SD: 2.0; 95%CI: 2.0 to 3.0), with a median of 2 and a range of 1 to 14 (Fig. 3). Of the 57 (90.5%) submissions for which information about the types of studies was available, 47 (74.6.7%) had at least one double-blind randomized clinical trial (RCT) as the highest level of evidence, 3 submissions (4.7%) had at least one open-label RCT as the highest level of evidence, and 7 (11.1%) had at least one non-controlled trial as highest level of evidence (Fig. 4).

**Fig. 5:**
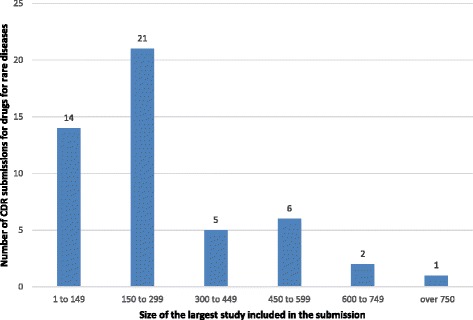
Number of CDR submissions by the size of the largest clinical study considered in each submission. Numbers above columns indicate the number of submissions

The study size (i.e., number of study subjects) for clinical studies that were considered in the CDR review of DRD submissions was reported for 49 (77.8%) of the included submissions. The largest clinical study per submission ranged from 20 to 742 subjects, with a median of 122 subjects. The average size of the largest study within each submission was 171.9 (SD: 171.9; 95%CI: 140.1 to 238.8). Figure 5 presents the number of submissions by the size of the largest included trial.

**Fig. 6:**
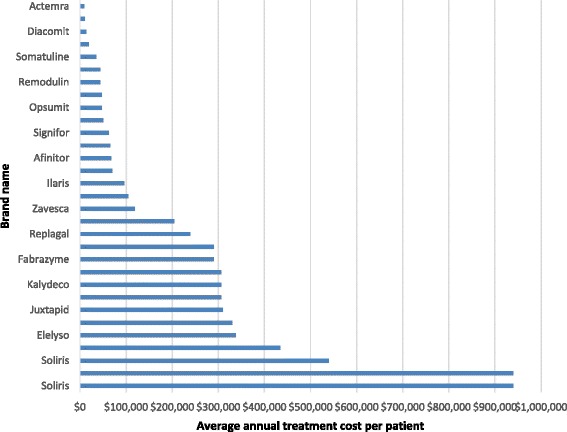
Average annual treatment cost per patient of drugs for rare diseases

Information regarding the annual treatment cost per patient was available for only 31 (49.2%) of the included DRD submissions. The reasons for not reporting the annual treatment cost included confidential pricing (15 submissions or 23.8%), an annual treatment cost not applicable to the indication under review (12 submissions or 19.0%), or ongoing or withdrawn submissions (5 submissions or 7.9%). The average annual treatment cost per patient for the 31 DRD submission for which cost information was available was $215,632 (SD: 240,858.8; 95%CI: 127,283.9 to 303,979.3), with a median of $104,890 with a minimum annual price of $9,706 and a maximum annual price of $940,084. Figure 6 illustrates almost half of the DRD submissions (45.2%) were for treatments with a cost greater than $200,000 per patient per year.

**Fig. 7:**
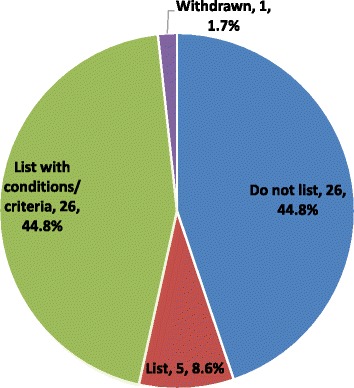
Listing reimbursement recommendations since 2004

**Fig. 8:**
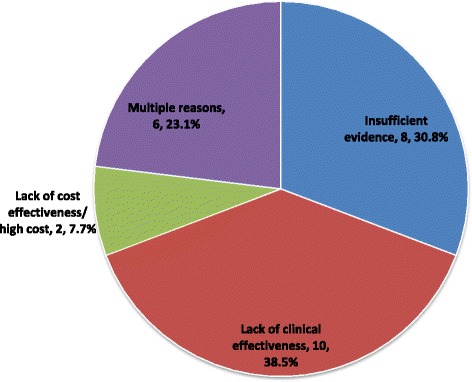
Reasons for negative reimbursement recommendation since 2004

Analysis of the type of reimbursement recommendations issued since 2004 for DRD submissions revealed that a negative reimbursement recommendation was issued for 26 (44.8%) submissions, while one submission was withdrawn (1.7%); the remaining 31 submissions (53.5%) received positive reimbursement recommendations, of which 8.6% were to “list” and 44.8% were to “list with criteria/conditions” (Fig. 7). Not included in the previous analysis are four submissions that were ongoing at the time of data collection, as such no recommendation was available, and one submission was a request for advice that did not yield a published recommendation. Overall, reasons for a negative reimbursement recommendation included lack of clinical effectiveness (10 submissions or 38.5%), insufficient evidence (8 submissions or 30.8%), multiple reasons (6 submissions or 23.1%), and lack of cost-effectiveness/high cost (2 submissions or 7.7%) (Fig. 8). A subgroup analysis of reimbursement recommendations and reasons for a negative reimbursement recommendations issued after the new CDEC framework took effect in November, 2012, revealed that 15 submissions (65.2%) received a positive recommendation (“list with criteria/conditions”) while 8 submissions (34.8)% received a negative recommendation (“do not list”). Reimbursement recommendations. The most frequent reason for a negative reimbursement recommendation was insufficient evidence to determine clinical effectiveness (5 [62.5%] submissions), followed by lack of clinical effectiveness (2 [25.0%] submissions), and multiple reasons (1 [12.5%] submission). No submission received a negative reimbursement recommendation due to cost alone.

## Discussion

Since its inception in 2004, the CADTH CDR has received a total of 63 DRD submissions (based on a definition of a rare disease being any disease with a prevalence of <50 patients per 100,000). These 63 submissions have addressed 35 unique rare diseases, which represents a very small proportion of the approximately 7,000 rare diseases described to date [9]. The number of DRD submissions received annually increased recently, from 1 to 5 submissions between 2004 and 2006 to 8 to 10 submissions between 2013 and 2015. Whether this reflects a general increase in the development of DRDs in unclear, although this would not be an unreasonable conclusion based on reports by others of increased focus of manufacturers on treatment for rare diseases [1, 10]. If the increase in the number of DRD submissions observed in our study continues, this suggests that HTA agencies such as the CADTH CDR will likely be required to review an increasingly large number of DRD submissions in future, which could pose some challenges given the unique clinical and economic complexities of these types of submission.

One of the greatest challenges in determining whether treatments for rare diseases are effective is the generation of sufficiently robust clinical data, because it is difficult to adequately power clinical studies due to a dearth of patients with the rare disease being studied. The difficulties encountered with recruiting adequate numbers of patients has led to the general perception that many aspects of conventional study designs are unfeasible in the study of DRDs. We expected to find mostly small size studies and single arm uncontrolled designs. However, 82.5% of the submissions with information on study design included at least one double-blinded RCT, and 71.4% of the submissions had enrolled more than 150 patients in their studies; this was despite most DRD submissions being for DRDs with a prevalence of <10 patients per 100,000 people. Our findings provide evidence that the aforementioned perception may be incorrect in most cases, and instead, suggest that it is indeed possible to design and conduct high-quality clinical studies despite issues with patient recruitment.

The average annual treatment cost per patient for the DRD submissions made to the CADTH CDR was $215,631, and almost half of the reported treatment costs were over $200,000 annually. This average, however, was calculated from only approximately half of the submissions, as cost information was either not reported or was redacted from published reports. Cost information was not available in most cases due to confidential pricing. Although rare diseases affect small numbers of people on an individual basis, approximately 7,000 unique rare diseases have been described to date [7], and as a whole, it is estimated that rare diseases affect 1 in 12 Canadians [11]. Moreover, the pace of development of treatments for rare diseases has increased in recent years [1, 10], which might be reflected in the increased growth in the number of DRD submissions made to CDR over the last few years. The potential future implications on public financial health resources that could result from increased development and funding of high-cost DRDs will require the timely development of innovative regulatory, HTA, and reimbursement frameworks to mitigate issues such as high opportunity costs and to ensure access to effective DRDs.

Over half of all the DRD CDR submissions received a positive reimbursement recommendation. The ‘do not list’ reimbursement recommendation was mostly due to reasons related to either lack of clinical efficacy or lack of enough evidence to inform on clinical efficacy. Given that most studies have supplied high-quality design, the reason for a ‘do not list’ due to ‘lack of clinical effectiveness’ was largely concerned with a lack of clinically relevant outcomes. This can be a challenge in rare-disease research as the use of surrogate outcomes could prove more convenient, statistically and financially, than clinical outcomes. With the recent emphasis towards more engagement of patients, evaluation committees want to assess beyond the surrogate outcomes and need to know if tangible changes that will better patient’s quality of life are considered in the research [11]. Clinical outcomes, patient-reported outcomes with a minimally important clinical difference, and surrogate outcomes with established clinical associations are needed to address the insufficient evidence in some of the DRD CDR submissions.

A CDEC reimbursement recommendation is non-binding on the CDR-participating drug plans. Each of the drug plans subsequently makes its own decisions based on the CDEC recommendation in addition to other factors including the plan’s mandate, jurisdictional priorities, pan-Canadian Pharmaceutical Alliance (pCPA) negotiations, and financial resources. Although we used a prevalence rate of < 50 per 100,000 people as a definition of a rare disease for the purpose of this analysis, CADTH has no formal definition for DRDs. In March 2016, CADTH published an update to the CDEC framework [12]. The framework provides guidance on the deliberation for drugs that may fulfill a significant unmet clinical need, and the rarity of the condition is only one of several factors that are considered to define a significant unmet clinical need.

While we have provided a comprehensive picture of the characteristics of DRD submissions to the CADTH CDR, our study is not without limitations. First, data extraction was conducted by one reviewer and verified by another, rather than in duplicate; while verification would ensure a high level of accuracy, it is not as accurate as a duplicate extraction. Second, our estimated prevalence values were in some cases derived from different sources and most were based on prevalence rates for populations in countries other than Canada; therefore, these values might not be an accurate representation of Canadian prevalence rates. In addition, we did not take into account the variability in prevalence among different locations; therefore, we might have included diseases that are not rare in all countries, while we might not have included diseases that are rare in some countries. This issue was further complicated by the fact that a challenge for the reviewers was whether to consider a drug that targets a small subpopulation of a common disease as a DRD. It was decided to keep the focus on drugs with a condition that is unique and rare versus a very small subpopulation of a relatively more common disease. Finally, the number of submissions that we identified was not sufficiently large to perform robust statistical analysis of associations among different characteristics.

## Conclusions

The annual number of DRD submissions made to the CADTH CDR has approximately doubled increased since 2013 compared to previous years, reflecting growth in the development of treatments for rare diseases. Over the last decade, more than half of DRD submissions resulted in a positive CDEC reimbursement recommendation, while most submissions that received a negative recommendation were characterized by low-quality clinical evidence and/or a lack of supportive clinical evidence. Although the average cost of DRD treatments in submissions made to the CDR is high, high cost was not a reason for a negative reimbursement recommendation in any recommendation after October 2012.

## Abbreviations

CADTH: Canadian Agency for Drugs and Technologies in Health
CDEC: Canadian Drug Expert Committee
CDR: Common Drug Review
DRD: Drugs for rare diseases
HTA: Health technology assessment
MCID: Minimal clinically important difference
pCODR: Pan-Canadian Oncology Drug Review
QALY: Quality-adjusted life year
